# Three new species of the primitively segmented spider genus *Songthela* (Araneae, Mesothelae) from Guizhou Province, China

**DOI:** 10.3897/zookeys.1037.62916

**Published:** 2021-05-13

**Authors:** Zhaoyang Chen, Dengqing Li, Daiqin Li, Xin Xu

**Affiliations:** 1 College of Life Sciences, Hunan Normal University, Changsha 410081, Hunan Province, China Hunan Normal Unviersity Changsha China; 2 Department of Biological Sciences, National University of Singapore, 14 Science Drive 4, 117543, Singapore National University of Singapore Singapore Singapore

**Keywords:** COI, Heptathelinae, Liphistiidae, morphology, taxonomy, trapdoor spiders

## Abstract

We diagnose and describe three new species of the primitively segmented spider genus *Songthela* from Guizhou Province, China, based on morphological characters and molecular data: *S. liui***sp. nov.** (♂♀), *S. tianzhu***sp. nov.** (♂♀), and *S. yuping***sp. nov.** (♂♀). We provide the genetic distances within and among the three new species based on the DNA barcode gene, cytochrome c oxidase subunit I (COI) to support our descriptions. We also provide the COI GenBank accession codes for the three new species for future identification.

## Introduction

**Figure 1.**
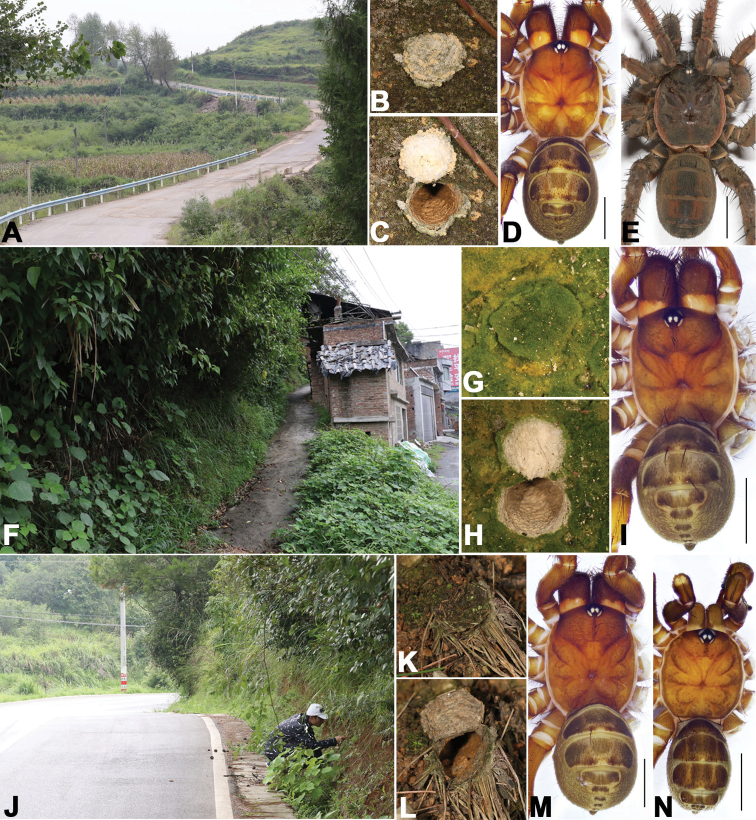
Microhabitat and general somatic morphology of three new *Songthela* species **A–E***Songthela liui* sp. nov.: **D** XUX–2018–381, female **E** XUX–2018–375A, male **F–I***Songthela tianzhu* sp. nov.: **I** XUX–2018–339, female **J–N***Songthela yuping* sp. nov.: **M** XUX–2018–380, female **N** XUX–2018–380A, male **A, F, J** microhabitat **B, C, G, H, K, L** the trapdoor with the door closed and open **D, E, I, M, N** dorsal view. Scale bars: 3 mm (**D, E, I, M, N**).

Liphistiidae is the only living family of the suborder Mesothelae. As the sister lineage to all other extant spiders, it retains several unique plesiomorphies, such as abdominal tergites (Fig. 1D, E, I, M, N) and spinnerets projected from the middle of the ventral abdomen (Haupt 2003; Schwendinger and Ono 2011; Xu et al. 2015a). Liphistiid spiders live in their underground borrow, which is closed by a silk-based trapdoor. The family contains 142 species belonging to eight genera in two subfamilies, Heptathelinae and Liphistiinae (World Spider Catalog 2021). Heptathelinae is confined to East Asia, while Liphistiinae is restricted to Southeast Asia (World Spider Catalog 2021). Liphistiinae contains the single genus *Liphistius* Schiödte, 1849, while Heptathelinae comprises the other seven genera (Xu et al. 2015a, b, 2021; World Spider Catalog 2021).

The genus *Songthela* includes 16 described species, 15 of which are distributed in southern China, and the remaining species, *S. sapana* (Ono, 2010), is found in northern Vietnam (World Spider Catalog 2021). Out of the 15 species from southern China, only one species, *S. pluma* Yu, Li & Zhang, 2018, is described from Guizhou Province. In this study, we diagnose and describe three new *Songthela* species from Guizhou Province based on both male and female genital morphology. To support our descriptions, we provide estimations of the intraspecific and interspecific genetical distances within and among the three new species as well as the interspecific genetic distances between the new species and other *Songthela* species based on the animal barcoding gene, cytochrome c oxidase subunit I (COI). For future identification, we also provide the GenBank accession codes of the COI for the three species.

## Material and methods

**Figure 2.**
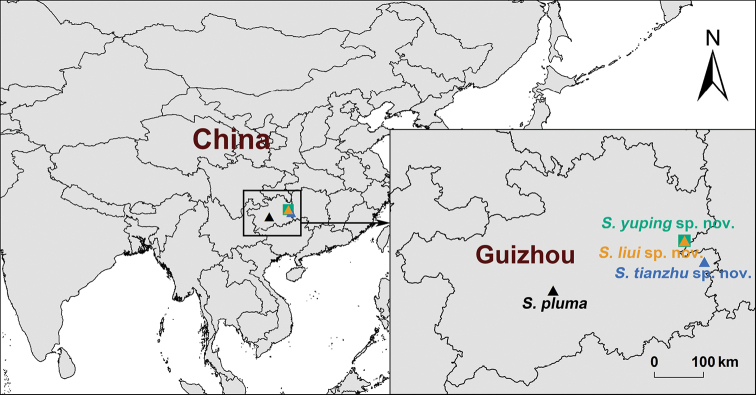
Map showing the type localities of the four *Songthela* species that are distributed in Guizhou Province, China.

All specimens were collected from Guizhou Province, China (Fig. 2). We took the subadults back to the laboratory and reared them until they reached adulthood. We removed the right four legs of adults, preserved them in 100% ethanol and kept them at –80 °C for molecular work. We preserved the remains of each specimen in 80% ethanol for morphological examination. All the type and voucher specimens are deposited at the College of Life Sciences, Hunan Normal University (**HNU**), Changsha, Hunan Province, China.

We examined and dissected the specimens using an Olympus SZ51 stereomicroscope. The soft tissues of female genitalia were degraded using 10 mg/ml trypsase (Bomei Biotech Company, Hefei, Anhui, China) for at least three hours at room temperature. We photographed male and female genitalia using a CCD digital camera mounted on an Olympus BX53 compound microscope, and then generated compound focused images using Helicon Focus v6.7.1. All measurements were made using a digital camera MC170HD mounted on a Leica M205C stereomicroscope and given in millimeters. Leg and palp measurements are given in the following order: leg total length (femur + patella + tibia + metatarsus + tarsus), palp total length (femur + patella + tibia + tarsus).

Abbreviations used: ALE = anterior lateral eyes; AME = anterior median eyes; BL = body length; CL = carapace length; Co = conductor; CT = contrategulum; CW = carapace width; E = embolus; OL = opisthosoma length; OW = opisthosoma width; PC = paracymbium; PLE = posterior lateral eyes; PME = posterior median eyes; RC = receptacular cluster; T = tegulum.

We extracted the total genomic DNA from spider legs using the Animal Genomic DNA Isolation Kit (Kangwei Biotech, Beijing, China). We used the primer pair LCO1490/HCO2198 (Folmer et al. 1994) to amplify COI. We used the following PCR reaction protocol: initial denaturation at 95 °C for 5 min; 35 cycles of denaturation at 95 °C for 1 min, annealing at 40 °C for 1 min, and elongation at 72 °C for 30 s; and final extension at 72 °C for 7 min (Xu et al. 2015c). The 25 μl PCR reaction contained 12.5 μl of 2×Taq MasterMix (KangWei Biotech, Beijing, China), 1 μl of each forward and reverse 10 μM primer, 1 μl of genomic DNA, and 9.5 μl of double-distilled H_2_O. The PCR products were visualized by agarose gel electrophoresis (1% agarose). All PCR products were purified and sequenced at Tsingke Biotechnology Company (Changsha, China).

Since only five known species (*S. goulouensis*, *S. huangyang*, *S. pyriformis*, *S. shuyuan* and *S. xiangnan*) have sequence data available from the holotype specimen (making the identification unambiguous), we obtained the publicly available COI sequences for these five species from the GenBank (for their GenBank accession codes, see Table 1) for comparison.

**Table 1.** Genetic distances within and among the three new species in this study and among the three new species and five known species based on COI sequences (K2P/*p*-distance). The GenBank accession codes of the known species and the number of specimens of each new species that were used to calculate the genetic distances are provided in parentheses. The specimen code and the GenBank accession code of the new species are provided in the descriptions.

	*S. liui* sp. nov. (*N* = 9)	*S. tianzhu* sp. nov. (*N* = 21)	*S. yuping* sp. nov. (*N* = 9)	*S. goulouensis*	*S. huangyang*	*S. pyriformis*	*S. shuyuan*
*S. liui* sp. nov.	0.1/0.1%	–	–	–	–	–	–
*S. tianzhu* sp. nov.	18.4/16.2%	0.9/0.9%	–	–	–	–	–
*S. yuping* sp. nov.	17.3/15.3%	6.6/6.2%	0/0%	–	–	–	–
*S. goulouensis* (MT102211)	14.4/12.9%	23.4/19.8%	21.5/18.5%	–	–	–	–
*S. huangyang* (MT102213)	19.8/17.3%	20.2/17.6%	21.1/18.2%	23.0/19.5%	–	–	–
*S. pyriformis* (MN400625)	17.7/15.6%	18.0/15.9%	17.3/15.3%	17.8/15.8%	19.2/16.8%	–	–
*S. shuyuan* (MN400635)	12.6/11.4%	19.6/17.1%	19.7/17.1%	16.5/14.4%	18.8/16.5%	17.9/15.8%	–
*S. xiangnan* (MT102212)	19.5/17.0%	14.2/12.7%	11.2/10.3%	23.3/19.7%	20.0/17.4%	19.7/17.3%	20.2/17.4%

We estimated intraspecific and interspecific genetic distances based on COI sequences using Kimura 2-parameter (K2P) and *p*-distance substitution models with MEGA v.6 (Tamura et al. 2013).

## Taxonomy

### Genus Songthela

Taxon classificationAnimaliaAraneaeLiphistiidae

Ono, 2000

85C58CB6-CA15-5AB0-A6EC-A6E567D8FDC9

#### Type species.

*Heptathela hangzhouensis* Chen, Zhang & Zhu, 1981

#### Diagnosis.

**Figure 3.**
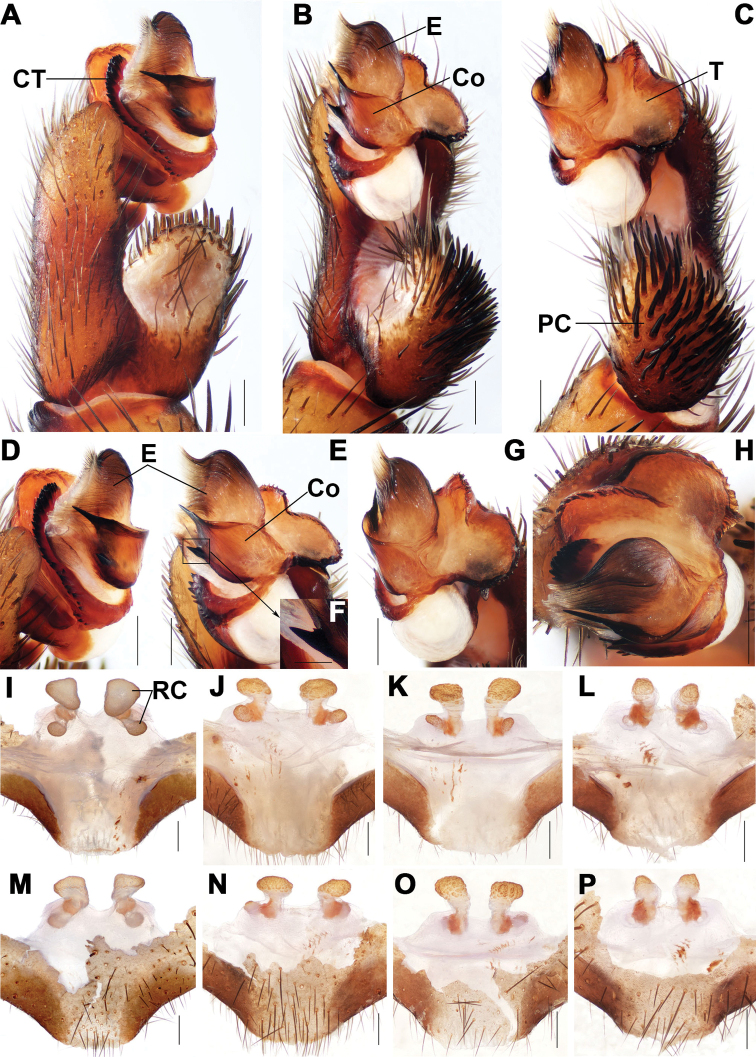
Male and female genital anatomy of *Songthela liui* sp. nov. **A, D** palp prolateral view **B, E** palp ventral view **C, G** palp retrolateral view **H** palp distal view **I–L** vulva dorsal view **M–P** vulva ventral view **A–H** XUX–2018–375A (holotype) **I, M** XUX–2018–381 **J, N** XUX– 2018–383A **K, O** XUX–2018–387D **L, P** XUX–2018–387. Scale bars: 0.3 mm (**A–E, G–P**); 0.1 mm (**F**).

**Figure 4.**
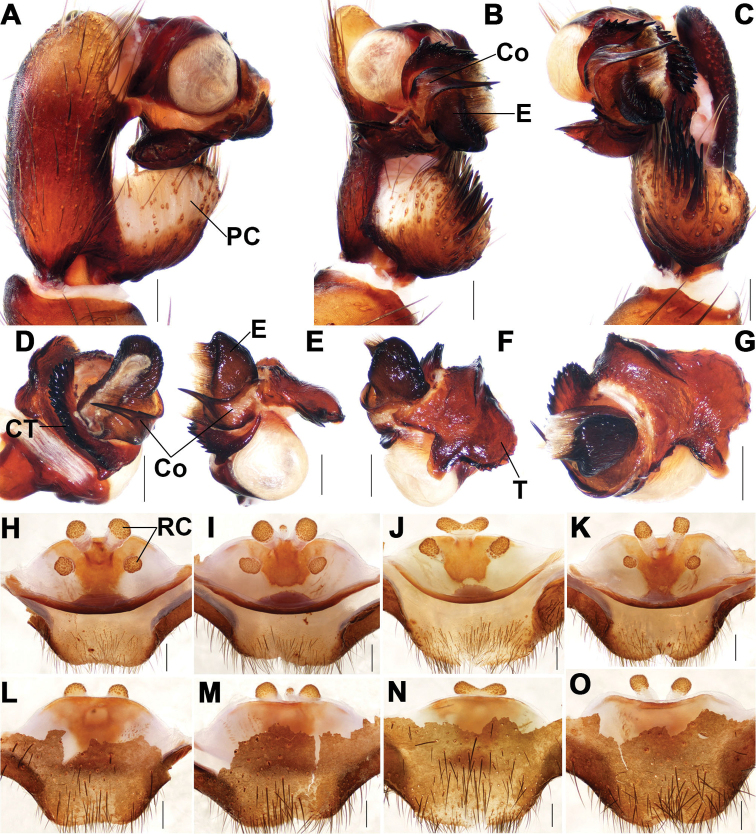
Male and female genital anatomy of *Songthela tianzhu* sp. nov. **A, D** palp prolateral view **B, E** palp ventral view **C, F** palp retrolateral view **G** palp distal view **H–K** vulva dorsal view **L–O** vulva ventral view **A–G** XUX–2018–340A (holotype) **H, L** XUX–2018–339 **I, M** XUX–2018–338 **J, N** XUX–2018–340 **K, O** XUX–2018–341. Scale bars: 0.3 mm.

**Figure 5.**
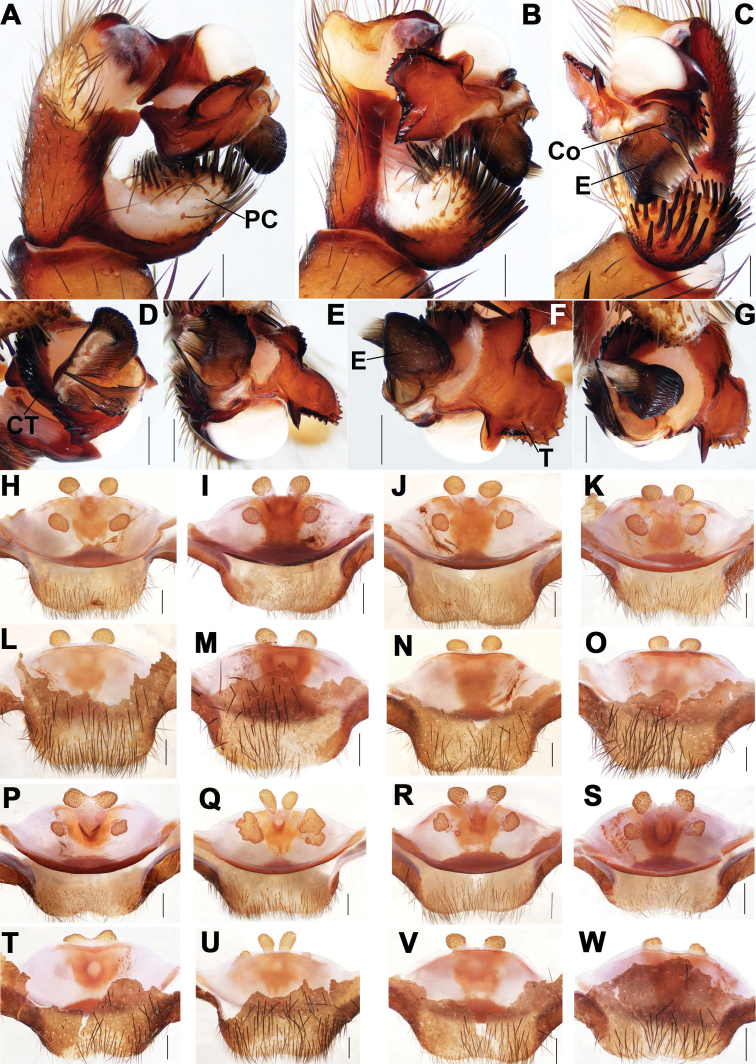
Male and female genital anatomy of *Songthela yuping* sp. nov. **A, D** palp prolateral view **B, E** palp ventral view **C, F** palp retrolateral view **G** palp distal view **H–K** vulva dorsal view **L–O** vulva ventral view **A–G** XUX–2018–380A (holotype) **H, L** XUX–2018–380 **I, M** XUX– 2018–374 **J, N** XUX–2018–377 **K, O** XUX–2018–382 **P, T** XUX–2018–382A **Q, U** XUX–2018–375 **R, V** XUX–2018–379 **S, W** XUX–2018–373. Scale bars: 0.3 mm.

*Songthela* males can be distinguished from those of all other Heptathelinae genera by the contrategulum with serrated edges (Figs 3A, D, G, 4B, D, G, 5C, D, G); by the proximal portion of the conductor relatively narrow and smooth, the distal portion gradually narrowed into one long apical spine (Figs 4D, E, 5D, E) or two apical spines (Fig. 3A, B, D, E); and by the distal portion of the embolus slightly sclerotized with a wide and flat opening (Figs 3A, D, 4B, D, 5C, D). *Songthela* females differ from those of all other Heptathelinae genera by four receptacular clusters separated from each other, the median pair situated along the anterior margin of the bursa copulatrix or close to the dorsal wall of the bursa copulatrix with distinct tubular stalks, the lateral ones located dorsolaterally (Figs 3I–P, 4H–O, 5H–W).

#### Composition.

*Songthela bristowei* (Gertsch, 1967), *S. ciliensis* (Yin, Tang & Xu, 2003), *S. goulouensis* (Yin, 2001), *S. hangzhouensis* (Chen, Zhang & Zhu, 1981), *S. huangyang* Li, Liu, Li & Xu, 2020, *S. jianganensis* (Chen, Gao, Zhu & Luo, 1988), *S. mangshan* (Bao, Yin & Xu, 2003), *S. pluma* Yu, Li & Zhang, 2018, *S. pyriformis* Li, Liu & Xu, 2019, *S. sapana* (Ono, 2010), *S. shei* (Xu & Yin, 2001), *S. shuyuan* Li, Liu & Xu, 2019, *S. wosanensis* (Wang & Jiao, 1995), *S. xiangnan* Li, Liu, Li & Xu, 2020, *S. xianningensis* (Yin, Tang, Zhao & Chen, 2002), *S. yunnanensis* (Song & Haupt, 1984).

#### Distribution.

Southern China (Guizhou, Hubei, Hunan, Sichuan, Yunnan, Zhejiang Provinces) and northern Vietnam (Lao Cai Province).

### Songthela liui sp. nov.

Taxon classificationAnimaliaAraneaeLiphistiidae

DB8F10A3-1C6D-585D-9827-41886FF5992F

http://zoobank.org/D485CADB-3AC6-49BA-BF06-805D00DB9390

Figure 3

#### Type material.

***Holotype*:** China · 1 ♂; Guizhou Province, Tongren City, Yuping Autonomous County, Zhujiachang Town, Yutang Village; 27.30°N, 108.89°E; alt. 542 m; 17 August 2018; D. Li, F.X. Liu, X. Xu, D.Q. Li and L. Yu leg.; XUX–2018–375A (matured on 5 May 2019 at HNU). ***Paratypes***: China · 1 ♀; same data as for the holotype; XUX–2018–381 · 9 ♀♀; Guizhou Province, Qiandongnan Autonomous Prefecture, Cengong County, Xiajiaao Village; 27.46°N, 108.83°E; alt. 552–553 m; 17 August 2018; D. Li, F.X. Liu, X. Xu, D.Q. Li and L. Yu leg.; XUX–2018–383, 383A, 385, 386, 387, 387A, 387C, 387D, 387E.

#### Diagnosis.

Male of *S. liui* sp. nov. resembles that of *S. hangzhouensis*, but can be distinguished from the latter by the base of the lower spine of the conductor wider and with a small spur (Fig. 3A, B, D–F), and by the tegulum with a small terminal apophysis (Fig. 3B, C, E, G, H); from that of *S. goulouensis* by the conductor with a shorter upper spine and the base of the lower spine of the conductor wider with a small spur (Fig. 3A, B, D–F), and by the marginal tegular apophysis with a slightly helicoid edge (Fig. 3B, C, E, G, H); from that of *S. shuyuan* by the contrategulum with an apophysis proximally (Fig. 3A, D), and by the base of the lower spine of the conductor wider with a small spur (Fig. 3B, E, F); from that of *S. yuping* sp. nov. by the conductor with two apical spines (Fig. 3A, B, D, E), by the contrategulum with smaller marginal teeth (Fig. 3A, D); from those of other *Songthela* species by the conductor with two conspicuous apical spines (Fig. 3A, B, D, E). Females of *S. liui* sp. nov. can be distinguished from those of *S. hangzhouensis* by the bases of the median receptacular clusters separated from each other (Fig. 3I–P); from those of *S. shuyuan* by the median receptacular clusters with shorter stalks (Fig. 3I–P); from those of *S. yuping* sp. nov. by the middle receptacular clusters situated at anterior margin of the bursa copulatrix, and distinctly larger than the lateral ones, and by the middle genital stalks separated from each other basally (Fig. 3I–P); and from those of other *Songthela* species by the middle receptacular clusters larger than the lateral ones and the bases of the middle ones close to the lateral ones (Fig. 3I–P).

#### Description.

**Male** (holotype; Fig. 1E). Carapace dark reddish brown, opisthosoma slightly reddish brown, with 12 dark reddish brown tergites, close to each other, 2–6 larger than others, and the 4^th^ largest; sternum narrow, much longer than wide; a few pointed hairs running over ocular area; chelicerae robust with promargin of cheliceral groove with 9 denticles of variable size; legs with hairs and spines; 7 spinnerets. Measurements: BL 14.25, CL 6.33, CW 5.21, OL 6.78, OW 4.62; ALE > PLE > PME > AME; leg I 18.44 (5.26 + 2.44 + 4.01 + 4.40 + 2.33), leg II 18.95 (4.72 + 2.54 + 4.02 + 4.95 + 2.72), leg III 21.33 (5.10 + 2.22 + 4.48 + 6.48 + 3.05), leg IV 27.85 (6.44 + 2.64 + 5.65 + 9.24 + 3.88).

***Palp*.** Paracymbium with numerous setae and spines at the tip, with an apophysis ventrally (Fig. 3B). Contrategulum with a small apophysis and three teeth proximally, the marginal teeth arranged sparsely and gradually split into two edges distally (Fig. 3A, D). The marginal tegular apophysis and the dorsal extension of terminal tegular apophysis with helicoid edges, and with a small triangular terminal tegular apophysis retrolaterally (Fig. 3B, C, E, G, H). Conductor smooth, fused with embolus basally, with two apical spines and a spur at the base of the lower spine from ventral view (Fig. 3A, B, D–F). Embolus with a flat opening distally and numerous ribbed ridges in middle and distal portion (Fig. 3A–E, G).

**Female** (XUX–2018–381; Fig. 1D). Carapace dark reddish brown, opisthosoma slightly brown, with 12 dark brown tergites, close to each other, 2–6 larger than others, and the 4^th^ largest; sternum narrow, much longer than wide; a few pointed hairs running over ocular area; chelicerae robust with promargin of cheliceral groove with 13 denticles of variable size; legs with hairs and spines; 7 spinnerets. Measurements: BL 14.97, CL 6.81, CW 5.66, OL 7.17, OW 5.13; ALE > PLE > PME > AME; palp 10.96 (3.67 + 1.81 + 2.46 + 3.02), leg I 13.70 (4.08 + 2.33 + 2.79 + 2.74 + 1.76), leg II 13.40 (4.01 + 2.28 + 2.45 + 2.79 + 1.87), leg III 14.66 (4.17 + 2.28 + 2.73 + 3.39 + 2.09), leg IV 20.35 (5.57 + 2.74 + 3.72 + 5.57 + 2.75).

***Female genitalia*.** Two pairs of receptacular clusters with tubular stalks. The middle pair of receptacular clusters situated at anterior margin of bursa copulatrix, separated from each other, larger than the lateral ones. The lateral ones ellipsoidal, situated dorsolaterally with short genital stalks. The bases of the middle receptacular clusters close to those of the lateral ones (Fig. 3I–P).

#### Variation.

Females vary in body size. The range of measurements as follows (*N* = 10): BL 10.51–14.76, CL 4.84–6.24, CW 4.08–5.95, OL 4.71–7.39, OW 3.86–5.48. The number of promargin of cheliceral groove varies from 11–13 (*N* = 10). There are 7 or 8 spinnerets. Moreover, female genitalia are somewhat variable: the median pair of receptacular clusters are different in shape, mushroom-like (Fig. 3J, K, N, O), triangular (Fig. 3I, M), or ovoid (Fig. 3L, P); the genital stalks of the middle receptacular clusters slightly vary in length (Fig. 3I–P).

#### Etymology.

The specific name is dedicated to Mr Fengxiang Liu for his kind instructions on all our collection.

#### Distribution.

Guizhou (Tianzhu, Cengong) Province, China.

#### GenBank accession number.

Holotype, XUX–2018–375A: MW450989; Paratypes, XUX–2018–383: MW808998; XUX–2018–383A: MW808999; XUX–2018–385: MW809000; XUX–2018–386: MW809001; XUX–2018–387: MW809002; XUX–2018–387A: MW809003; XUX–2018–387B: MW809004; XUX–2018–381: MW809005.

#### Remarks.

Although liphistiid spiders are known to have a high level of endemism with the increasing number of our collected liphistiid specimens, we have found more than one species in a few localities and also a few widespread species in the genus *Songthela* (unpublished data). In this study, we diagnosed two new *Songthela* species, *S. liui* sp. nov. and *S. yuping* sp. nov., after examining the specimens collected from Yutang Village, Zhujiachang Town, Yuping Autonomous County, Tongren City, Guizhou Province, based not only on male and female genital morphology, but also the genetic distances of COI. We provide the intraspecific genetic distances of *S. liui* sp. nov., and the interspecific genetic distances among the three new species, as well as among the new species and other known species (*S. goulouensis*, *S. huangyang*, *S. pyriformis*, *S. shuyuan* and *S. xiangnan*) (Table 1). The interspecific genetic distances were estimated based on the holotype of each species, except for *S. goulouensis*, which was based on the publicly available COI sequence from the GenBank along with its descriptions (Li et al. 2019, 2020).

### Songthela tianzhu sp. nov.

Taxon classificationAnimaliaAraneaeLiphistiidae

6ED18311-1FC4-5F1E-B2D5-02CC71524ED8

http://zoobank.org/9430C186-894D-4A77-ACDC-2C1955F2216B

Figure 4

#### Type material.

***Holotype*:** China · 1 ♂; Guizhou Province, Qiandongnan Autonomous Prefecture, Tianzhu County, Qinxiang Village; 26.92°N, 109.26°E; alt. 380 m; 16 August 2018; D. Li, F.X. Liu, X. Xu, D.Q. Li and L. Yu leg.; XUX–2018–340A (matured on 10 September 2019 at HNU). ***Paratypes***: China · 10 ♀♀; same data as for holotype; XUX–2018–336, 336A, 337, 338, 339, 340, 340B, 341, 345, 345A · 17 ♀♀; Guizhou Province, Qiandongnan Autonomous Prefecture, Tianzhu County, Mixi Village; 26.94°N, 109.08°E; alt. 543–552 m; 16 August 2018; D. Li, F.X. Liu, X. Xu, D.Q. Li and L. Yu leg.; XUX–2018–346, 346A, 347, 348, 349, 350, 351, 352, 353, 354, 354A, 354B, 355, 356, 356A, 357, 357A.

#### Diagnosis.

Male of *S. tianzhu* sp. nov. resembles that of *S. ciliensis*, but can be distinguished from the latter by the apical spine of the conductor with a spinule basally (Fig. 4D, E), and by the embolus with a curved margin ventrally (Fig. 4B, E, G); from those of other *Songthela* species by the smooth conductor with an apical spine, and the apical spine with a spinule basally (Fig. 4D, E). Females of *S. tianzhu* sp. nov. resemble those of *S. pluma* and *S. yuping* sp. nov., but can be distinguished from those of *S. pluma* by the stalks of the median pair receptacular clusters fused together basally (Fig. 4H–K); from those of *S. yuping* sp. nov. by the trapeziform genital area and the shallower depressions (Fig. 4H–O); from those of other *Songthela* species by two pairs of receptacular clusters situated on the dorsal wall of the bursa copulatrix (Fig. 4H–O).

#### Description.

**Male** (holotype). Carapace yellow brown; opisthosoma light brown, with 12 dark brown tergites, close to each other, 2–6 larger than others, and the 4^th^ largest; sternum narrow, much longer than wide; a few pointed hairs running over ocular area; chelicerae robust with promargin of cheliceral groove with 9 denticles of variable size; legs with sturdy hairs and spines; 7 spinnerets. Measurements: BL 12.16, CL 5.77, CW 5.02, OL 5.56, OW 3.50; ALE > PLE > PME > AME; leg I 18.02 (5.11 + 2.28 + 3.73 + 4.57 + 2.33), leg II 17.09 (4.63 + 1.88 + 3.18 + 5.08 + 2.32), leg III 19.40 (4.59 + 1.96 + 3.90 + 5.76 + 3.19), leg IV 25.91 (6.29 + 2.50 + 5.23 + 8.05 + 3.84).

***Palp*.** Paracymbium unpigmented and unsclerotized in prolateral view, with several setae and spines on the tip (Fig. 4A–C). Contrategulum with a distinct apophysis on the proximal portion and a regular dentate edge (Fig. 4D, E). Tegulum with a slightly dentate marginal apophysis and the dorsal extension of the terminal apophysis, and with a thumb-like terminal tegular apophysis retrolaterally (Fig. 4F, G). Conductor with a wide base and fused with embolus, the distal portion gradually narrow to a long apical spine with a spinule basally (Fig. 4D, E). Embolus with a flat opening distally, numerous ribbed ridges in middle and distal portion, and with a curved margin ventrally (Fig. 4B–G).

**Female** (XUX–2018–339; Fig. 1I). Carapace dark reddish brown; opisthosoma light brown, with 12 dark brown tergites, close to each other, 2–6 larger than others, and the 4^th^ largest; sternum narrow, much longer than wide; a few pointed hairs running over ocular area; chelicerae robust with promargin of cheliceral groove with 10 denticles of variable size; legs with sturdy hairs and spines; 7 spinnerets. Measurements: BL 11.68, CL 5.52, CW 4.81, OL 5.40, OW 4.33; ALE > PLE > PME > AME; palp 10.32 (3.45 + 1.74 + 2.19 + 2.66), leg I 11.28 (3.62 + 1.98 + 2.20 + 2.03 + 1.45), leg II 10.90 (3.62 + 1.98 + 2.20 + 2.03 + 1.45), leg III 12.09 (3.55 + 2.09 + 2.22 + 2.47 + 1.76), leg IV 17.92 (5.02 + 2.52 + 3.53 + 4.53 + 2.32).

***Female genitalia*.** Two pairs of ovoid receptacular clusters with tubular stalks, situated on the dorsal wall of the bursa copulatrix. The median pair slightly larger than (Fig. 4H, I, K) or similar to (Fig. 4J) the lateral ones, and the bases of the middle stalks fused together (Fig. 4H–K).

#### Variation.

Females vary in body size. The range of measurements as follows (*N* = 27): BL 10–13.82, CL 4.57–6.94, CW 4.14–6.10, OL 4.54–6.57, OW 3.77–6.00. The number of promargin of cheliceral groove varies from 10–13 (*N* = 27). There are 7 or 8 spinnerets. In addition, female genitalia show somewhat intraspecific variation: there is an additional receptacular cluster situated at the middle of the median pair (Fig. 4I, M); the genitalia stalks of the median pair are fused together basally (Fig. 4H, I, K) or the middle receptacular clusters are fused together (Fig. 4J); some specimens have slightly longer middle genital stalks than others (Fig. 4K).

#### Etymology.

The species epithet, a noun in apposition, refers to the type locality.

#### Distribution.

Guizhou (Tianzhu), China.

#### GenBank accession number.

Holotype, XUX–2018–340A: MW450988; Paratypes, XUX–2018–336: MW809006; XUX–2018–337: MW809007; XUX–2018–338: MW809008; XUX–2018–339: MW809009; XUX–2018–340: MW809010; XUX–2018–341: MW809011; XUX–2018–342: MW809012; XUX–2018–343: MW809013; XUX–2018–345: MW809014; XUX–2018–346: MW809015; XUX–2018–347: MW809016; XUX–2018–348: MW809017; XUX–2018–349: MW809018; XUX–2018–350: MW809019; XUX–2018–352: MW809020; XUX–2018–353: MW809021; XUX–2018–354: MW809022; XUX–2018–355: MW809023; XUX–2018–356: MW809024; XUX–2018–357: MW809025.

#### Remarks.

The intraspecific genetic distances of the new species and the interspecific genetic distances between the new species and the other two new species and five known species are shown in Table 1.

### Songthela yuping sp. nov.

Taxon classificationAnimaliaAraneaeLiphistiidae

572C55C6-3C36-504A-8909-88377D8F5D60

http://zoobank.org/1B725706-AC1A-4B8D-8F90-B5AE5FC920E1

Figure 5

#### Type material.

***Holotype*:** China · 1 ♂; Guizhou Province, Tongren City, Yuping Autonomous County, Zhujiachang Town, Yutang Village; 27.30°N, 108.89°E; alt. 546 m; 17 August 2018; D. Li, F.X. Liu, X. Xu, D.Q. Li and L. Yu leg.; XUX–2018–380A. ***Paratypes***: China · 10 ♀♀; same data for the holotype; XUX–2018–373, 374, 375, 376, 377, 378, 379, 380, 382, 382A.

#### Diagnosis.

Male of *S. yuping* sp. nov. resembles that of *S. pluma* and *S. xiangnan*, but can be distinguished from that of *S. pluma* by the apical spine of the conductor with a spinule basally (Fig. 5C, D, E), the contrategulum with fewer teeth (Fig. 5D); from that of *S. liui* sp. nov. by the conductor with only a long apical spine, and the middle part of conductor covered with numerous small spines (Fig. 5C–E); from that of *S. xiangnan* by the blade-shaped spine of the conductor with one tip (Fig. 5C–E), by the semielliptical contrategulum (Fig. 5D, G), and by the tegulum with a small terminal apophysis retrolaterally (Fig. 5B, F); from that of other *Songthela* species by the conductor covered with several short spines on the middle part (Fig. 5C–E). Females of *S. yuping* sp. nov. resemble those of *S. pluma* and *S. tianzhu* sp. nov., but can be distinguished from those of *S. pluma* by the arc-shaped anterior margin of the bursa copulatrix (Fig. 5H–K, P–S); from those of *S. tianzhu* sp. nov. by the slightly longer middle genital stalks (Fig. 5H–W), and by the rectangular genital area and the slightly deeper depressions (Fig. 5H–K, P–S); from those of *S. liui* sp. nov. by the two pairs of receptacular clusters situated on the dorsal wall of the bursa copulatrix, and the middle genital stalks fused together basally (Fig. 5H–W); from those of other *Songthela* species by four receptacular clusters situated on the dorsal wall of the bursa copulatrix (Fig. 5H–W).

#### Description.

**Male** (holotype; Fig. 1N). Carapace yellow brown, opisthosoma slightly brown, with 12 dark brown tergites, close to each other, 2–6 larger than others, and the 4^th^ largest; sternum narrow, much longer than wide; a few pointed hairs running over ocular area; chelicerae robust with promargin of cheliceral groove with 9 denticles of variable size; legs with sturdy hairs and spines; 7 spinnerets. Measurements: BL 9.65, CL 4.53, CW 3.62, OL 4.63, OW 3.28; ALE > PLE > PME > AME; leg I 15.40 (4.42 + 2.00 + 3.43 + 3.73 + 1.82), leg II 14.06 (4.15 + 1.85 + 3.48 + 2.90 + 1.68), leg III 17.08 (4.26 + 1.88 + 3.38 + 5.25 + 2.31), leg IV 23.05 (5.66 + 2.18 + 4.59 + 7.37 + 3.25).

***Palp*.** Paracymbium with numerous setae and spines at the distal portion (Fig. 5A). Contrategulum with an irregular dentate edge and an apophysis proximally (Fig. 5D). Tegulum with a serrated marginal apophysis and a dorsal extension of terminal apophysis, and with a small thumb-like terminal tegular apophysis retrolaterally (Fig. 5B, F, G). Conductor fused with embolus basally, covered with several small spines in the middle part, and a long blade-shaped apical spine with a spinule basally (Fig. 5C, D, E). Embolus with a flat opening in distal portion and numerous ribbed ridges in middle and distal portion (Fig. 5C–G).

**Female** (XUX–2018–382A; Fig. 1M). Carapace yellow brown, opisthosoma slightly brown, with 12 dark brown tergites, close to each other, 2–6 larger than others, and the 4^th^ largest; sternum narrow, much longer than wide; a few pointed hairs running over ocular area; chelicerae robust with promargin of cheliceral groove with 12 denticles of variable size; legs with sturdy hairs and spines; 7 spinnerets. Measurements: BL 13.67, CL 6.60, CW 5.43, OL 6.28, OW 5.06; ALE > PLE > PME > AME; palp 11.50 (3.75 + 2.05 + 2.42 + 3.28), leg I 13.37 (4.41 + 2.32 + 2.53 + 2.57 + 1.54), leg II 13.00 (3.85 + 2.30 + 2.27 + 2.79 + 1.79), leg III 13.21 (3.95 + 2.42 + 2.09 + 2.94 + 1.81), leg IV 19.08 (5.35 + 2.64 + 3.35 + 5.38 + 2.36).

***Female genitalia*.** Two pairs of receptacular clusters with tubular stalks, situated on the dorsal wall of the bursa copulatrix. The median ones similar to or slightly larger than the lateral ones, with smooth genital stalks and fused together basally (Fig. 5H–W).

#### Variation.

Females vary in body size. The range of measurements as follows (*N* = 10): BL 9.65–14.14, CL 4.53–6.60, CW 3.62–5.68, OL 4.59–6.71, OW 3.28–5.30. The number of promargin of cheliceral groove varies from 9–12 (*N* = 10). Moreover, the female genitalia show somewhat intraspecific variation: the genital stalks of the median receptacular clusters are different in shape, either “Y” shaped (Fig. 5K, Q, R, S), “V” (Fig. 5H–J), or fused together (Fig. 5P); the lateral receptacular clusters are irregular, and larger than the median ones (Fig. 5Q).

#### Etymology.

The species epithet, a noun in apposition, refers to the type locality.

#### Distribution.

Guizhou (Yuping), China.

#### GenBank accession number.

Holotype, XUX–2018–380A: MW450990; Paratypes, XUX–2018–373: MW809026; XUX–2018–374: MW809027; XUX–2018–375: MW809028; XUX–2018–376: MW809029; XUX–2018–377: MW809030; XUX–2018–378: MW809031; XUX–2018–379: MW809032; XUX–2018–380: MW809033.

#### Remarks.

The intraspecific genetic distances of *S. yuping* sp. nov., and the interspecific genetic distances between *S. yuping* sp. nov. and the other two new species and the five known species are shown in Table 1.

## Supplementary Material

XML Treatment for Songthela

XML Treatment for Songthela liui

XML Treatment for Songthela tianzhu

XML Treatment for Songthela yuping
